# Dressing interventions to heal pressure ulcers: A protocol for an overview of systematic reviews and meta-analysis

**DOI:** 10.1097/MD.0000000000022699

**Published:** 2020-10-09

**Authors:** Jie Geng, Yali Zhao, Zheyuan Wang, Mancai Wang, Zhihong Wei

**Affiliations:** Lanzhou University Second Hospital, Lanzhou University, Lanzhou, Gansu, People's Republic of China.

**Keywords:** dressing, meta-analysis, methodological quality, overview, pressure ulcer, systematic reviews

## Abstract

**Background:**

Pressure ulcer (PU) is defined as a lesion or trauma to the skin and underlying tissue resulting from unrelieved pressure, shear, friction, moisture, or a combination of all these, usually appearing over a bony prominence. We aim to evaluate the credibility of systematic reviews and meta-analyses that assess the effectiveness, safety, and economy of the dressing treatments for PU through an overview.

**Methods:**

We searched the following electronic bibliographic databases: PubMed, Embase, Cochrane Library, CINAHL Complete, PsycARTICLES, PsycINFO, DynaMed Plus, as well as the Chinese databases without any language restriction. We will include meta-analyses that dressings treatments in the management of PUs. For each meta-analysis, we will estimate the effect size of a treatment through the random-effect model and the fixed-effect model, and we will evaluate between-study heterogeneity (Cochrane's *Q* and *I*^2^ statistics) and small-study effect (Egger's test); we will also estimate the evidence of excess significance bias. Methodological quality of each meta-analysis will be evaluated by using Assessment of Multiple Systematic Reviews 2.

**Results:**

This study is ongoing and the results will be submitted to a peer-reviewed journal for publication.

**Ethics and dissemination:**

Ethical approval is not applicable, since this is an overview based on published articles.

**Protocol registration number:**

The protocol has been registered on PROSPERO under the number CRD42020161232.

## 1 Introduction

Pressure ulcers are local damage to the skin and/or underlying tissue due to unreleased pressure or pressure caused by shear or friction.^[1,2]^ It is usually located at the site of the bone protrusion or related to medical equipment.^[3,4]^ It can be manifested as intact skin or open ulcers, which may be accompanied by pain and even lead to conflicts between doctors and patients.^[5–7]^ In the evaluation process of nursing quality, the incidence of pressure ulcer is one of the key indicators.^[8,9]^ Studies in recent years have shown that the incidence and prevalence of pressure ulcers have been high. Pressure ulcers are common clinical chronic refractory wounds and one of the most common complications in hospitalized patients, due to their high incidence and prevalence, long treatment cycles, and high cost of treatment, the prevention, and treatment of pressure ulcers has been widely concerned.^[10,11]^

A multi-center clinical observation study in the United Kingdom found that the prevalence of pressure ulcers in the community can be as high as 7.7%, even after removing nursing home patients with a higher prevalence of pressure ulcers, the prevalence is as high as 3.8%.^[12]^ Multiple research results show that the overall incidence of pressure ulcers abroad is 7.1% to 23.8%.^[13–15]^ The occurrence of pressure ulcers prolongs the length of hospital stay by three times and is associated with complications or even the risk of death.^[6,16]^ Although there are differences in the methods of statistics on the incidence and prevalence of pressure ulcers, there is no doubt that patients with pressure ulcers have formed a relatively large group, and their clinical treatment is relatively difficult, the treatment cycle is long, and the cost is high. Bringing a heavy financial burden to patients and society.^[10,17,18]^ The annual cost of treatment for pressure ulcers in the United Kingdom is about 1.4 to 2.1 billion pounds (accounting for 4% of the national medical cost).^[19]^ The cost in the United States is about $11 billion.^[11]^

Therefore, finding a reliable treatment method for pressure ulcers is one of the priorities of clinical nursing workers. Local treatment methods for pressure injuries mainly focus on promoting wound healing through the use of various types of wound dressings. Dressings can help wound debridement, reduce bacterial load and prevent further trauma. At present, the types of dressings used to treat pressure injuries mainly include new types of dressings, wet dressings, silver ion dressings, foam dressings, hydrocolloid dressings, seaweed salt dressings, and other wound dressings.^[20,21]^ Nonetheless, there is a lack of evidence to help us decide which dressing is clinically cost-effective.^[22,23]^

Systematic reviews based on strictly designed high-quality randomized controlled trials can provide scientific evidence for health decisions and can also form higher-level recommendations in the guidelines.^[20,24,25]^ However, empirical research shows that there are a large number of unnecessary, misleading and conflicting Systematic review and meta-analysis.^[26,27]^ Therefore, this study overview meta-analysis and systematic reviews to evaluate the effectiveness, safety, and economy of various dressings for the treatment of stress injuries, with a view to providing clinical nursing workers with an effective basis for the treatment of stress injuries.

## 2 Methods and analysis

### 2.1 Study registration

This protocol has been registered on the international prospective register of a systematic review (PROSPERO) (https://www.crd.york.ac.uk/PROSPERO/#myprospero), and the registration number is CRD42020161232.

### 2.2 Study inclusion and exclusion criteria

#### 2.2.1 Types of studies

Inclusion: Systematic reviews. Reviews will be considered systematic if they meet the four following criteria:

1. searches at least one database;
2. reports their selection criteria;
3. provides a list and
4. synthesis of included studies.

Exclusion:

1. Editorials
2. Commentaries

#### 2.2.2 Types of participants

We will include trials with participants of any age described as having a pressure ulcer (bed sore, pressure sore, or decubitus ulcer) of stage 2 to 4 and in any setting. Studies were also excluded if they included other types of wounds (e.g., chronic wound and venous leg ulcers).

#### 2.2.3 Types of interventions

Various dressings for pressure ulcer treatment (e.g., saline gauze, hydrocolloid dressings, hydrogel dressings, moisture-retentive dressings, and foam dressing).

#### 2.2.4 Types of outcomes measures

Main outcomes: Time to complete healing/rate of healing (as defined in the trial), the proportion of wounds completely healed in a specified period time (as defined in the trial), pressure injury occurrence reported adverse events.

Additional outcomes: length of hospital stays, incidence of different type of infection(s).

### 2.3 Search strategy

#### 2.3.1 Electronic searches

We will search the following electronic bibliographic databases: PubMed (inception-present), Embase (inception-present), Cochrane Library (inception-present), CINAHL Complete (inception-present), PsycARTICLES (inception-present), PsycINFO (inception-present), DynaMed Plus (inception-present), as well as the Chinese databases.

#### 2.3.2 Other resources

Searches of the grey literature, and the bibliographies of relevant papers were also used to complement the results of the database searches.

#### 2.3.3 Search strategies

All databases will be based on the MeSH and text word search and will be adjusted according to the specific database, take PubMed as an example, the search strategy is shown in Table 1

**Table 1:**
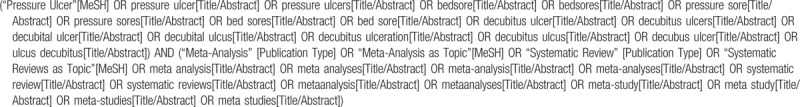
The search strategy take Pubmed as an example.

#### 2.3.4 Literature screening

All search results are imported into EndNote X8 literature management software, two reviewers (JG, YLZ) will screen the titles and abstracts of literature independently, then read the full text to assess literature according to the inclusion and exclusion criteria, any disagreements will be resolved by a third reviewer (YS).

#### 2.3.5 Data extraction

We, the reviewers (ZYW), will extract information from the included studies using an extraction sheet. We will contact the corresponding authors for any missing information. We will extract the maximum amount of available data from the available publications. Disagreements will be resolved by discussion and with a third review author, if needed.

Independently, two review authors will extract the following data:

Author, country, and publication year of study, setting of study (e.g., primary care, hospital), dressings, participants, interventions and comparators, included study designs, exclusion criteria, quality assessment, method(s) of synthesis, number of included studies, summary of results and conclusions, and outcome.

### 2.4 Study quality assessment

Two review authors will independently assess the risk of bias in included reviews by using the AMSTAR 2 tool for quality assessment of systematic reviews. The AMSTAR 2 tool is a modified version of AMSTAR that fits more closely the systematic reviews that include both RCTs and NRCTs. Two reviewers (MCW and ZYW) will rate the quality of each meta-analysis as high, moderate, low, and critically low based on the overall score of the AMSTAR2. Conflicts between reviewers will be resolved through discussion and involving experts.

### 2.5 Statistical analysis

#### 2.5.1 Data synthesis

A descriptive synthesis of assessed systematic reviews is planned. The effect sizes from the meta-analyses will be presented as mean differences (WMD), standardized mean differences (SMD), odds ratios (OR), relative risks (RR), or risk differences (RD), depending on the data reported by the authors. In addition, whenever possible, the results will be reported with 95% confidence intervals (95% CI). Excluded papers will be listed, with reasons for exclusion stated.

#### 2.5.2 Assessment of heterogeneity

We can reflect the feasibility of meta-analysis by evaluating the heterogeneity of the included studies. According to the guideline of Cochrane Handbook, heterogeneity between RCTs can be quantified using I-square (*I*^2^) values, if *I*^2^ is >50%, significant heterogeneity is considered, then a subgroup analysis is needed to determine the source of heterogeneity. If there is missing data in the included study, we will contact the author by email or phone to get the missing data. We will use Egger's test to evaluate publication bias and small-study effect, and a *P* value < .1 in the test confirms the bias and small-study effect.

#### 2.5.3 Subgroup analysis

If the evidence is sufficient, we will conduct a subgroup analysis to determine the difference between different basic illness, over 60 years old and <60 years old, pressure ulcers in different stages, etc.

### 2.6 Quality of evidence

Two reviewers (JG, YLZ) will use the GRADE (Grading of Recommendations Assessment, Development and Evaluation) method to assess the quality of evidence of included studies. The evidence levels classified into four levels: high, moderate, low, or very low.

## 3 Discussion

The occurrence of pressure ulcers is becoming more and more universal, which has a great negative impact on individuals, families, and society. It is increasingly important to find effective and economical treatments. Dressing treatments have proved to be effective, but different dressings have different advantages and disadvantages. Overall, this overview will be the first to assess the impact of different dressings in treating pressure ulcer. The results of this overview may provide practical guidance for the clinic and provide new research ideas for researchers.

## Author contributions

**Conceptualization:** Zhihong Wei, Mancai Wang.

**Data curation:** Jie Geng, Zhihong Wei.

**Formal analysis:** Jie Geng, Zhihong Wei.

**Funding acquisition:** Jie Geng, Zheyuan Wang.

**Methodology:** Zheyuan Wang.

**Software:** Yali Zhao, Mancai Wang.

**Writing – original draft:** Jie Geng, Zhihong Wei, Yali Zhao, Zheyuan Wang, Mancai Wang.

**Writing – review & editing:** Jie Geng, Zhihong Wei, Yali Zhao, Zheyuan Wang, Mancai Wang.

## Abbreviations

Abbreviations: AMSTAR = Assessment of Multiple Systematic Reviews, CI = confidence intervals, GRADE = Grading of Recommendations Assessment, Development and Evaluation, NRCT = nonrandomized controlled trials, PU = pressure ulcer, RCT = randomized controlled trials.
