# Dressing interventions to heal pressure ulcers: A protocol for an overview of systematic reviews and meta-analysis: Erratum

**DOI:** 10.1097/MD.0000000000023109

**Published:** 2020-11-06

This article, “Dressing interventions to heal pressure ulcers: A protocol for an overview of systematic reviews and meta-analysis”,^[1]^ which originally published in Volume 99, Issue 41 of *Medicine*, was originally published with an incorrect author list. The author liast has ben updated from Jie Geng, Yitong Cai, Yali Zhao, Zheyuan Wang, Mancai Wang, and Zhihong Wei to Jie Geng, Yali Zhao, Zheyuan Wang, Mancai Wang, and Zhihong Wei in the published version.
